# Time Course Analysis of the Effects of Botulinum Neurotoxin Type A on Pain and Vasomotor Responses Evoked by Glutamate Injection into Human Temporalis Muscles

**DOI:** 10.3390/toxins6020592

**Published:** 2014-02-10

**Authors:** Larissa Bittencourt da Silva, Dolarose Kulas, Ali Karshenas, Brian E. Cairns, Flemming W. Bach, Lars Arendt-Nielsen, Parisa Gazerani

**Affiliations:** 1 Center for Sensory-Motor Interaction (SMI), Department of Health Science and Technology, Aalborg University, Aalborg DK-9220, Denmark; E-Mails: larissa@hst.aau.dk (L.B.S.); demil07@student.aau.dk (D.K.); brcairns@mail.ubc.ca (B.E.C.); lan@hst.aau.dk (L.A.-N.); 2 Neurology Department, Aalborg University Hospital, Aalborg DK-9100, Denmark; E-Mails: ali.karshenas@rn.dk (A.K.); fwb@rn.dk (F.W.B.); 3 Faculty of Pharmaceutical Sciences, The University of British Columbia, 2405 Wesbrook Mall, Vancouver, British Columbia V6T 1Z3, Canada

**Keywords:** botulinum neurotoxin type A, temporalis muscle, glutamate, pain, neurogenic, vasomotor

## Abstract

The effect of botulinum neurotoxin type A **(**BoNTA) on glutamate-evoked temporalis muscle pain and vasomotor responses was investigated in healthy men and women over a 60 day time course. Subjects participated in a pre-BoNTA session where their responses to injection of glutamate (1 M, 0.2 mL) and saline (0.2 mL) into the temporalis muscles were assessed. On Day 1, BoNTA (5 U) was injected into one temporalis muscle and saline into the contralateral temporalis muscle, in a randomized order. Subjects then received intramuscular injections of glutamate (1 M, 0.2 mL) into the left and right temporalis muscles at 3 h and subsequently 7, 30 and 60 days post-injection of BoNTA. Pain intensity, pain area, and neurogenic inflammation (skin temperature and skin blood perfusion) were recorded. Prior to BoNTA treatment, glutamate evoked significantly greater pain and vasomotor reactions (*P* < 0.001) than saline. BoNTA significantly reduced glutamate-evoked pain intensity (*P* < 0.05), pain area (*P* < 0.01), skin blood perfusion (*P* < 0.05), and skin temperature (*P* < 0.001). The inhibitory effect of BoNTA was present at 3 h after injection, peaked after 7 days and returned to baseline by 60 days. Findings from the present study demonstrated a rapid action of BoNTA on glutamate-evoked pain and neurogenic inflammation, which is in line with animal studies.

## 1. Introduction

Botulinum neurotoxin type A (BoNTA) is used for several clinical conditions for its well-known muscle relaxation effect [1,2]. This compound also exerts analgesic effects where pain relief occurs prior to muscular relaxation or in areas without muscle hyperactivity [3,4]. This suggests that BoNTA-induced analgesia may occur by mechanisms distinct from muscle relaxation. Indeed, an example of this is the injection of BoNTA into craniofacial muscles as a prophylactic treatment to reduce the frequency of headaches in chronic migraine [4,5,6]. It has been suggested that this treatment is effective because BoNTA decreases peripheral input from pericranial muscles [3], which in turn reduces sensitization in the trigeminovascular system [7,8]. The temporalis muscle is one of the craniofacial muscles injected with BoNTA. Recently, it was reported the mechanical threshold of temporalis muscle nociceptors was significantly increased 3 h after injection of BoNTA into the temporalis muscles of female rats [9]. Over the same time course, BoNTA was found to significantly reduce the interstitial concentration of the excitatory amino acid glutamate in the temporalis muscle, which suggests that altered peripheral glutamatergic tone plays a role in the analgesic mechanism of BoNTA.

Injection of glutamate into human craniofacial muscles (e.g., masseter or temporalis muscles) evokes pain and has been used as an experimental human model of craniofacial muscle pain conditions, such as myofascial temporomandibular disorders [10,11,12,13,14,15,16]. Glutamate induces muscle pain, mechanical sensitization and neurogenic inflammation through the activation of peripheral glutamate receptors [9,10,11,12,17]. Glutamate-evoked muscle pain and nociceptor discharge is sexually dimorphic, with greater responses seen in females than in males [13,15]. In rats, BoNTA attenuated neurogenic inflammation (vasodilatation) and blocked nociceptor mechanical sensitization induced by injection of glutamate into the temporalis muscle within 3 h of its intramuscular injection [9]. However, as these experiments were performed under anesthesia, it is uncertain how rapid the effects of BoNTA appear or whether these effects contribute to altered pain sensitivity in humans. The current study investigated the effect of BoNTA on glutamate-evoked temporalis muscle pain and neurogenic vasodilation in healthy human subjects over a two month period. Based on animal findings, it was anticipated that BoNTA would reduce glutamate-induced vasomotor reactions in human temporalis muscle with a rapid onset, similar to that observed in animal experiments. The aims of the present study were to: (1) determine whether single administration of intramuscular BoNTA affects glutamate-induced pain intensity and vasomotor reactions in the temporalis muscles of healthy men and women; (2) explore the time course of BoNTA effects on these parameters; and (3) identify any sex-related differences in these responses.

## 2. Results and Discussion

### 2.1. Results

All volunteers finished the study and no safety issues were reported following the injections or applied assessment techniques.

#### 2.1.1. Glutamate Pain Model Validation

Administration of glutamate into the temporalis muscle of healthy humans induced pain and vasomotor responses and yielded a feasible human experimental pain model with no side effects.

##### Pain

**Figure 1:**
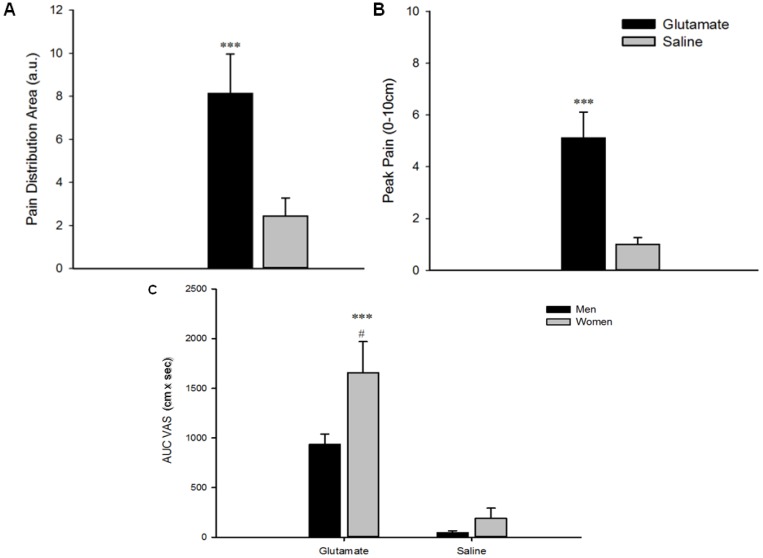
Glutamate-evoked pain characteristics. Pain characteristics following glutamate (1 M, 0.2 mL) and saline (0.9%, 0.2 mL) injections into the temporalis muscles. (**A**) Pain distribution area on face charts (a.u.); (**B**) Peak pain intensity (visual analogue scale (VAS) 0–10 cm); and (**C**) Area under the VAS curve (cm × sec). Data are shown as mean and SEM, ∗∗∗ Indicates that glutamate evoked a significantly higher response when compared with saline (*P* < 0.001) and # indicates that women had a greater response when compared to men (*P* < 0.05).

Intramuscular injection of glutamate provoked higher peak pain intensity (5.1 ± 0.45 cm on VAS 0–10 cm) compared with saline (1.0 ± 0.27 cm on VAS 0–10 cm) (F = 114.65, *P* < 0.001). The overall pain indicated by AUC VAS (cm × sec) was also larger following glutamate injection compared with saline (F = 66.78, *P* < 0.001). AUC VAS was influenced by sex (F = 4.85, *P* < 0.05) with women (AUC VAS: Glutamate = 1657 ± 232 cm × sec, Saline = 192 ± 74 cm × sec) reporting a significantly greater intensity of pain than men (AUC VAS: Glutamate = 935 ± 232 cm × sec, Saline = 47 ± 74 cm × sec). Pain drawing results showed that pain distribution area following glutamate injection (8.14 ± 1.84 a.u.) was perceived as larger than with the saline (2.44 ± 0.83 a.u.) (F = 19.13, *P* < 0.001). There was no sex-related difference in this measurement. Figure 1 shows all pain characteristics response from glutamate and saline injections.

##### Vasomotor Responses

**Figure 2:**
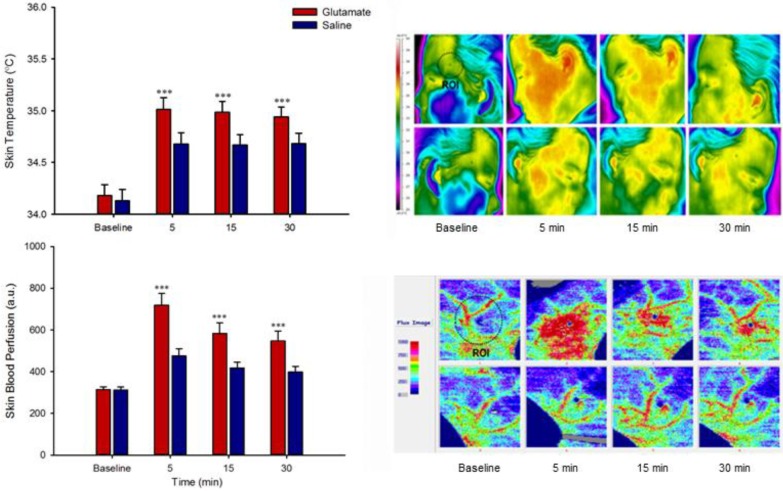
Glutamate-evoked vasomotor responses. Skin temperature (left, upper panel) and blood perfusion (left, lower panel) at baseline and 5, 15 and 30 min following intramuscular injection of glutamate (1 M, 0.2 mL), and saline (0.9%, 0.2 mL), into the temporalis muscle of healthy volunteers. Data are shown as mean and SEM, ∗∗∗ Indicates that glutamate injection evoked a higher vasomotor response (temperature and blood perfusion) when compared with saline (*P* < 0.001). Typical recordings have been shown in right upper and lower panels for skin temperature and blood flow, from baseline and up to 30 min after the injections (upper panel: glutamate; lower panel: saline). ROI: region of interest.

Local skin temperature (F = 36.33, *P* < 0.001) and blood perfusion (F = 32.14, *P* < 0.001) were elevated following glutamate injection in comparison with saline (Figure 2). Men had a higher mean blood perfusion response (F = 5.14, *P* < 0.05), while, women had a larger average skin temperature response (F = 7.16, *P* < 0.05).

#### 2.1.2. The Effect of BoNTA on Glutamate-Evoked Pain and Vasomotor Responses

Pre-treatment of temporalis muscle by BoNTA attenuated glutamate induced pain and vasomotor responses with no self-reported side effects.

##### Pain

**Figure 3:**
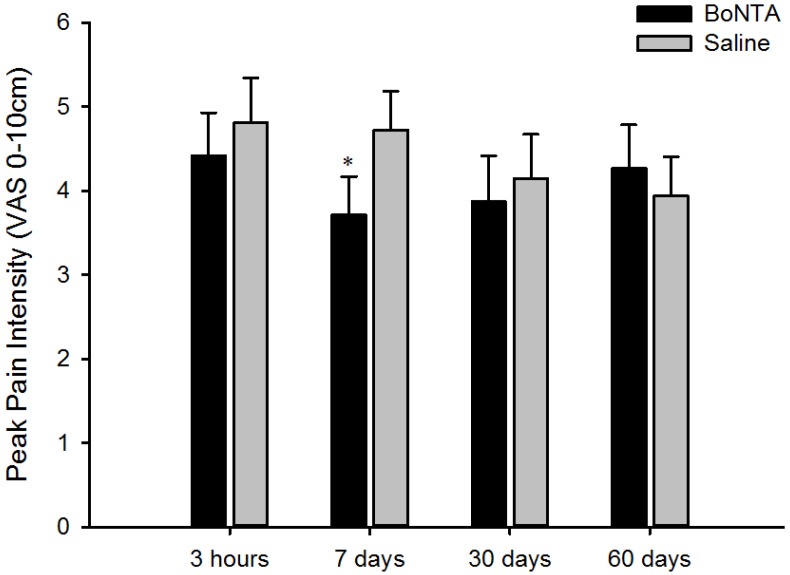
BoNTA’s effect on glutamate-evoked peak pain intensity. Peak pain intensity (VAS 0–10 cm) after glutamate (1 M, 0.2 mL) into the BoNTA (5 U, 0.1 mL) and saline (0.9% and 0.1 mL) pre-treated temporalis muscles of the healthy volunteers. Data are shown as mean and SEM. ∗ Indicates that on Day 7 the pain peak was significantly decreased on the BoNTA side when compared to saline (*P* < 0.05).

**Figure 4:**
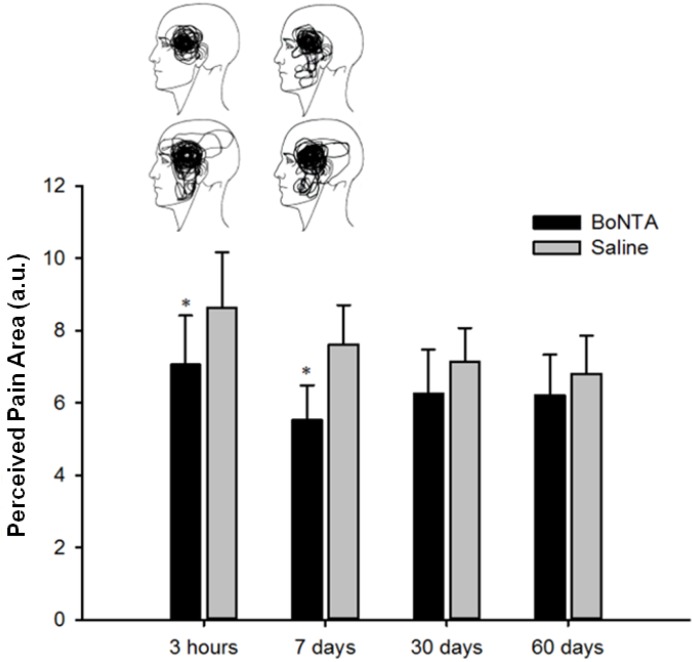
BoNTA’s effect on perceived pain area. Pain distribution areas following intramuscular injection of glutamate (1 M, 0.2 mL) into the temporalis muscle of healthy volunteers. Three hours and Days 7, 30 and 60 refer to the time after BoNTA (5 U, 0.1 mL) and saline (0.9%, 0.1 mL) injections. (Data are shown as mean and SEM, ∗ *P* < 0.05). Face charts illustrate 3 h and 7 days (left to right) after glutamate injections in the pre-treated BoNTA side (upper drawings) and saline side (lower drawings).

Glutamate-evoked peak pain intensity from BoNTA pre-treated muscle was lower than in saline pre-treated muscles (F = 4.180, *P* < 0.05). *Post hoc* analysis showed a significance difference in pain intensity on Day 7 only (Figure 3). Perceived pain area after glutamate injection was significantly smaller in muscle pre-treated with BoNTA compared with saline treated muscle (F = 10.602, *P* < 0.01). *Post hoc* analysis revealed a significant difference between BoNTA and saline pre-treated muscles at 3 h (F = 4.69, *P* < 0.05) and 7 days (F = 6.46, *P* < 0.05). Figure 4 shows a superimposition of the pain drawings for all subjects at two different time points. No sex related difference was found in any of the pain responses between BoNTA and saline pre-treated muscles. 

##### Vasomotor Responses

**Figure 5:**
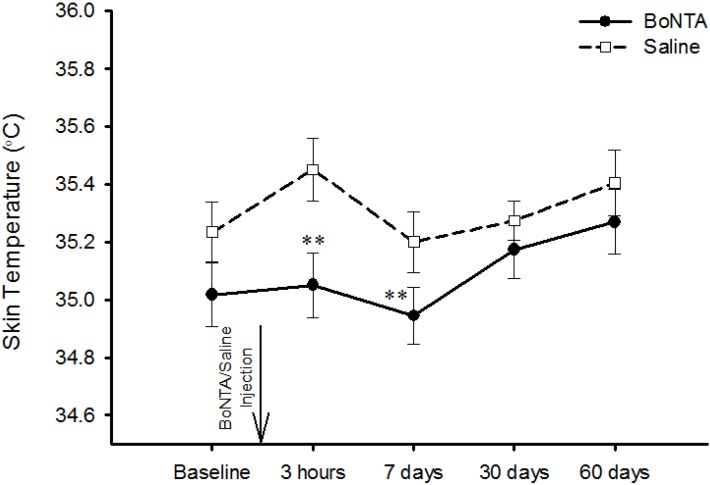
BoNTA’s effect on skin temperature. Skin temperature following intramuscular injection of glutamate (1 M, 0.2 mL) into the temporalis muscle of healthy volunteers. Three hours and 7, 30 and 60 days refer to the time after BoNTA and saline injections. ∗∗ Indicates a significant difference in skin temperature when BoNTA was compared with saline . (mean and SEM, ∗∗ *P* < 0.01: BoNTA *vs* Saline).

BoNTA reduced the increased skin temperature which was caused by glutamate injection (F = 18.729, *P* < 0.001). The comparison between the effect of BoNTA and saline on glutamate-evoked increases in skin temperature is shown in Figure 5. *Post hoc* analysis showed that significant side-side differences in temperature were found at 3 h (F = 8.78, *P* < 0.01) and 7 days (F = 11.13, *P* < 0.01) post BoNTA injection. BoNTA had a significantly greater inhibitory effect on glutamate-evoked increases in skin temperature in men than in women (F = 4.331, *P* < 0.05).

BoNTA also reduced the elevated blood perfusion which was caused by glutamate injection (F = 4.39, *P* < 0.05)*.* This effect was detectable at 3 h and 7 days following BoNTA pre-treatment (F = 6.50, *P* < 0.01). The reduction in glutamate-induced increased blood perfusion was greater in women than in men at 3 h (F = 4.88, *P* < 0.05) and 7 days (F = 5.91, *P* < 0.05) post injection. For details see Table 1.

**Table 1:** Summary of main outcomes after glutamate (1 M, 0.2 mL) injection into the BoNTA (5 U, 0.1 mL) and saline (0.9%, 0.1 mL) pre-treated temporalis muscles. Data are shown as mean and SEM. Please note that baseline measurements for temperature and blood flow were back to normal and no pain was reported before glutamate injection (data are not shown). The table shows values for skin temperature and blood perfusion, pain distribution area, peak pain intensity and area under the curve from the visual analogue scale [AUC VAS (cm × sec)]. Pooled, and data from males and females separately, are presented in order to show different outcomes from 3 h and 7, 30 and 60 days following BoNTA and saline pre-treatments.

Day	BoNTA				Saline			
	1 (3h)	7	30	60	1 (3h)	7	30	60
Temperature (°C) Mean ± SEM								
Pooled	35.23 ± 0.10*	34.94 ± 0.10*	35.17 ± 0.10	35.27 ± 0.11	35.45 ± 0.11*	35.20 ± 0.11*	35.27 ± 0.07	35.40 ± 0.11
Male	35.11 ± 0.16	34.91 ± 0.11	35.13 ± 0.13	35.2 ± 0.12	35.11 ± 0.14^#^	35.21 ± 0.12	35.26 ± 0.08	35.29 ± 0.14
Female	35.36 ± 0.13	34.98 ± 0.16	35.22 ± 0.16	35.36 ± 0.21	35.79 ± 0.12^#^	35.19 ± 0.18	35.28 ± 0.11	35.55 ± 0.18
Blood Perfusion (a.u.) Mean ± SEM								
Pooled	587 ± 55*	525 ± 44	571 ± 46	550 ± 56	663 ± 61*	578 ± 53	591 ± 46	549 ± 43
Male	705 ± 94^#^	623 ± 67^#^	580 ± 72	651 ± 95	753 ± 99	696 ± 90^#^	684 ± 77	641 ± 77^#^
Female	469 ± 42^#^	421 ± 46^#^	563 ± 60	449 ± 47	573 ± 64	460 ± 42^#^	498 ± 40	456 ± 22^#^
Perceived Pain Area (a.u.) Mean ± SEM								
Pooled	7.06 ± 1.36*	5.53 ± 0.96*	6.26 ± 1.21	6.22 ± 1.11	8.63 ± 1.53*	7.62 ± 1.09*	7.14 ± 0.93	6.81 ± 1.06
Male	5.73 ± 1.35	4.44 ± 1.13	4.43 ± 1.05	5.92 ± 1.60	8.46 ± 1.79	6.04 ± 1.03	7.24 ± 1.11	7.03 ± 1.04
Female	8.40 ± 2.35	6.62 ± 1.55	8.10 ± 2.12	6.51 ± 1.60	8.81 ± 2.54	9.19 ± 1.87	7.04 ± 1.53	6.58 ± 1.88
Peak Pain Intensity (VAS 0–10cm) Mean ± SEM								
Pooled	4.42 ± 0.51	3.71 ± 0.46*	3.87 ± 0.55	4.27 ± 0.51	4.81 ± 0.53	4.72 ± 0.46*	4.15 ± 0.52	3.94 ± 0.46
Male	3.89 ± 0.52	3.00 ± 0.55	2.90 ± 0.60	3.67 ± 0.57	4.41 ± 0.59	4.27 ± 0.53	3.50 ± 0.51	3.37 ± 0.51
Female	4.95 ± 0.88	4.42 ± 0.71	4.84 ± 0.86	4.87 ± 0.84	5.21 ± 0.89	5.18 ± 0.76	4.80 ± 0.91	4.52 ± 0.76
AUC VAS (cm × sec) Mean ± SEM								
Pooled	955 ± 151	800 ± 146	905 ± 189	847 ± 140	1066 ± 166	929 ± 127	966 ± 160	808 ± 131
Male	788 ± 152	637 ± 163	616 ± 181	659 ± 154	870 ± 181	844 ± 154	741 ± 171	715 ± 144
Female	1121 ± 260	963 ± 240	1194 ± 322	1034 ± 229	1262 ± 274	1014 ± 205	1191 ± 264	901 ± 222

Notes: * P < 0.05: BoNTA *vs* Saline (3-way repeated ANOVA, Bonferroni test); # P < 0.05 : Males *vs* Females (3-way repeated ANOVA, Bonferroni test).

### 2.2. Discussion

#### 2.2.1. Glutamate-Evoked Experimental Pain Model

The present study showed that intramuscular injection of glutamate into the temporalis muscle of healthy men and women evokes pain and vasomotor responses that are sexually dimorphic. Women reported greater pain intensity. In contrast, men had a higher mean blood perfusion response than women after injection of glutamate. A previous study did not find a sex-related difference in glutamate-evoked temporalis muscle pain intensity, but this study used a lower concentration of glutamate (500 mM) [18]. A number of reports have found, however, that glutamate (1000 or 500 mM) injection into the masseter muscle evokes pain of greater intensity in women than in men [13,15,18,19]. This is, however, the first report of vasomotor responses associated with temporalis muscle pain and sex-related responses.

Vasomotor responses have been investigated with a thermocamera after glutamate was injected into the rat temporalis muscle [9]. Injection of glutamate increased temporalis muscle temperature by ~1 °C; an indication that glutamate injection increased muscle blood flow [9,11]. This glutamate-evoked increase in temporalis muscle blood flow was attenuated with NMDA, neurokinin (NK)1 and calitonin gene related peptide (CGRP) receptor antagonists, which suggests that glutamate-induced vasodilation is mediated through activation of peripheral NMDA receptors and release of substance P and CGRP [9]. Since the temporalis muscle is a superficial muscle, with a lower thickness compared with muscles like masseter muscle, responses recorded at skin can reflect local changes in muscle blood flow. Women showed a higher skin temperature following the administration of glutamate or saline into the temporalis muscle. This could be due to differences in heat transfer from the temporalis muscle to the overlying skin due to variations in skin structure, collagen content, and/or diverse microvasculature in men and women [20,21]. A linear association between surface temperature and blood flow has not always been shown [22]. In contrast, men had a greater blood perfusion of the muscle after injection of glutamate, although the reason for this sex-related difference is not clear.

#### 2.2.2. The Effect of BoNTA

##### Pain

The temporalis muscle is one of the injection sites for BoNTA when it is used for chronic migraine headache prophylaxis [4,5,6]. It is proposed that injection of BoNTA in craniocervical muscles may reduce peripheral input from muscle nociceptors involved in triggering a headache [4,23,24]. However, it is still unknown whether this theory might be responsible for the analgesic effect of BoNTA. BoNTA significantly reduced areas of perceived pain after injection of glutamate both 3 h and 7 days post injection. It was also found that BoNTA could significantly reduce glutamate-evoked temporalis muscle peak pain one week after injection, but that a significant analgesic effect was no longer demonstrable 30 days after injection. However, even at Day 7, BoNTA reduced glutamate-evoked peak pain intensity by only about 20% in temporalis muscle. In contrast, co-administration of the NMDA receptor antagonist ketamine with glutamate reduces glutamate-evoked masticatory muscle pain by ~50% [11,18]. It has been proposed that the analgesic mechanism of BoNTA in muscle results from the blockade of the vesicular release of glutamate and neuropeptides, e.g., CGRP or substance P (SP), from the endings of muscle nociceptors through the cleavage of the vesicular docking protein SNAP-25 [4,6,8,9,25]. The use of a high concentration glutamate to evoke muscle pain in the present study would by-pass one of the key mechanisms thought to contribute to BoNTA-induced muscle analgesia, which involves the reduction of interstitial glutamate concentration [9,17]. Thus, the greater effect on pain spread and more modest reduction in peak glutamate-evoked muscle pain by BoNTA observed in the present study likely reflects the component of glutamate-evoked pain due to release of neuropeptides e.g., CGRP and SP. However, it has been proposed recently that the longer term effects of BoNTA may involve retrograde transport of the toxin to the level of the sensory ganglion [26]. If this does occur, it would be expected to attenuate the release of various neurotransmitters by both ganglion neurons and satellite glial cells. Recent evidence indicates that modulation of the concentration of glutamate in the ganglion alters nociceptor sensitivity [27,28], suggesting another possible mechanism that could contribute to the analgesic effect of BoNTA.

While the analgesic efficacy of BoNTA has been shown in several studies [29] there are studies where no analgesic effect was observed [30,31,32,33]. The design of most animal and human studies to investigate BoNTA effects on nociception at least one week after treatment has been based on the clinical observations where the effect of BoNTA is not obvious shortly after its administration [23,34]. The results of the present study agree with these previous findings, but also indicate that the pain model employed plays an important role in the detection of acute BoNTA-induced analgesia.

##### Vasomotor Responses

BoNTA not only reduced pain, but also reduced elevated skin temperature and blood perfusion caused by glutamate. A similar effect of BoNTA on vasomotor responses has been shown in a previous study in rats after injection of glutamate into the temporalis muscle and in a human experimental pain study investigating subcutaneous injection of capsaicin as the pain model [35,36,37]. In rats, co-administration of glutamate, NK1 or CGRP receptor antagonists attenuates the increases in muscle blood flow after intramuscular injection of glutamate [9,11]. These findings suggest that activation of peripheral glutamate receptors depolarizes the endings of small diameter afferent fibers leading to the release of the CGRP and substance P, which cause the vasodilation. In the present study, BoNTA significantly reduced glutamate-evoked increases in skin temperature and blood perfusion 3 h after its injection into the temporalis muscle. A similarly fast onset for inhibition of glutamate-induced vasodilatation was noted in the rat temporalis muscle [9]. In the current study, this inhibitory effect of BoNTA on neurogenic vasodilatation was maintained for at least 7 days in healthy human subjects, and coincided with the inhibition of pain in these subjects. This finding bolsters the argument that in this model, BoNTA is reducing the component of pain due to the release of neuropeptides. These findings add further support to the concept that thermal imaging of the muscle could be developed as a biomarker of muscle pain and/or the efficacy of analgesic agents in reducing this pain.

As mentioned, glutamate injections induced greater increases in skin temperature in women and greater increases in blood perfusion in men. Curiously, BoNTA had a significantly greater inhibitory effect on glutamate-evoked increases in skin temperature in men but its inhibitory effect on increases in blood perfusion was greater in women. It is unclear at present why sex-related differences in the effect of BoNTA were detected. As there were no differences between men and women with regard to the analgesic effect of BoNTA, there appears to be little if any functional significance of these changes, but this needs further investigation before drawing any firm conclusion.

## 3. Experimental Section

### 3.1. Materials and Methods

#### 3.1.1. Subjects

Thirty healthy subjects were recruited through on-campus advertisements at Aalborg University, Denmark. The group consisted of 15 men (mean age ± SEM: 23.6 ± 0.51 years) and 15 women (23.3 ± 0.62 years).

All subjects were screened by the study physician (AK) before participating in any experimental procedure. The screening session included a review of medical history as well as a general physical and neurological examination. Subjects were excluded if any previous or present systemic, skin or neuromuscular diseases were identified or a history of migraine or chronic tension type headache, severe allergy, alcohol or drug abuse was identified. None of the subjects had taken part in another investigational drug or device study within 30 days prior to the first session or throughout the study period. Female subjects were not pregnant or having irregularities in their menstrual cycles.

Participants were fully informed about the goal, procedures and safety aspects of the study before they gave a signed written informed consent prior to the start of the experiments. Subjects were asked to refrain from use of any medication, alcohol and caffeine at least 24 h before each experimental session.

#### 3.1.2. Study Design

**Figure 6:**
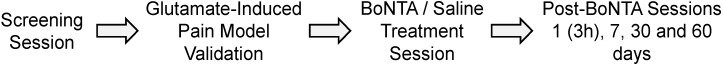
Study overview. Simplified experimental design showing visits and injections, starting with a screening session followed by experimental sessions up to 60 days.

**Figure 7:**
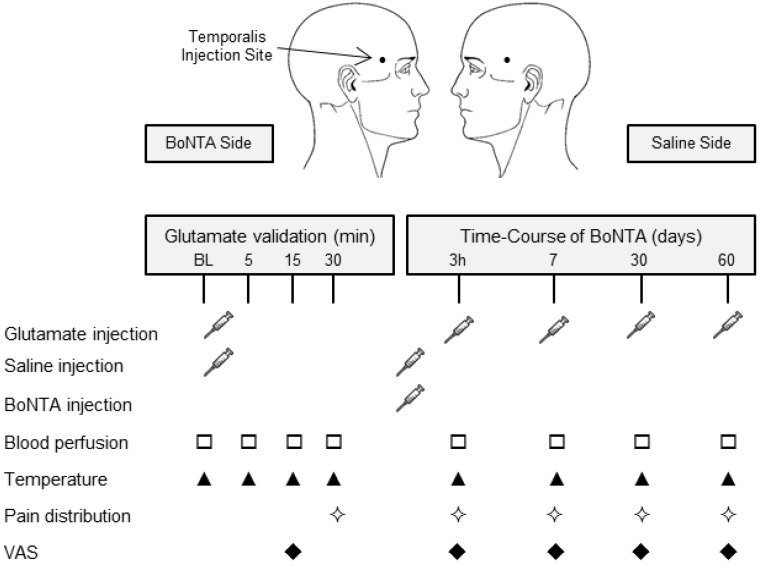
Protocol overview. Experimental protocol for interventions and measurements. (VAS: visual analogue scale).

A double blinded, randomized and placebo controlled study was designed and approved by the local ethics committee (Region of North Jutland, Denmark; N-20100105), and the Danish data protection agency. The study was carried out in accordance with the Good Clinical Practice (GCP) guidelines and the Declaration of Helsinki. Subjects participated in five sessions after the screening, including a pain model validation session (glutamate-induced experimental pain model), a treatment session (BoNTA and Saline) and four follow-up sessions (3 h and 7, 30 and 60 days following BoNTA). A diagram of the overall study design can be found in Figure 6. BoNTA injections were given at the Neurology Department, Aalborg Hospital, where all safety precautions were implemented. The same investigator (LBS) performed all tests at the pain laboratory of the Center for Sensory-Motor Interaction, Aalborg University, Denmark. Figure 7 summarizes sensory and vasomotor assessments performed in each experimental session.

#### 3.1.3. Glutamate Pain Model Validation Session

Sterile solutions of monosodium l-glutamate (MSG) (0.2 mL; 1 M; pH 7.0–7.2) (Aalborg and Skanderborg Hospital Pharmacies, Denmark) and isotonic saline (0.2 mL; 0.9%) were injected intramuscularly with single use tuberculin syringes fitted with 27-gauge disposable needles. Glutamate and isotonic saline (control) were injected into the right or left temporalis muscles with 30 min gap in between of the injections. The order and site of injection (left *versus* right) was randomized. The subjects were unaware of the content of the syringes. Prior to injections the skin was cleaned with alcohol and allowed to completely dry before needle insertion.

#### 3.1.4. BoNTA and Saline Injections

Each vial of BoNTA (BOTOX^®^, Allergan, Inc., Irvin, CA; 200 U/vial) was reconstituted with preservative-free 0.9% Sodium Chloride Injection, using one vial per subject, as recommended by the manufacturer. A single injection of BoNTA was made into the anterior temporalis muscle (5 U/0.1 mL). The same volume of sterile physiological saline (0.1 mL, 0.9%) was injected into the contralateral anterior temporalis muscle to serve as a vehicle control injection. The injection sides were randomized and both the experimenter and subjects were blinded to the content of the injections. Electromyographic (EMG)-guided injections (Clavis™, Medtronic, Copenhagen, Denmark) with disposable needle electrodes (Neuroline Injoject, 27G, Ambu A/S, Balerup, Denmark) were used in order to ensure intramuscular injection. The site of injections in the temporalis muscle were marked with ink and mapped on a human face chart printed on a transparent sheet to allow the re-identification of injection location for the subsequent sessions.

#### 3.1.5. Sensory and Vasomotor Assessments

##### Pain Characteristics

Glutamate-evoked pain was rated continuously by the subjects on an electronic visual analogue scale (VAS, Aalborg University). The pain rating was recorded for 10 minutes following the injections. The lower endpoint of the scale was marked “no pain” and the upper endpoint labeled “the most pain imaginable”. Peak pain intensity (the highest VAS score) and the area under VAS curve [AUC VAS (cm × sec)] were extracted from the VAS profiles using PINARQ software (Aalborg University). The outcome was stored in an excel data sheet and used for further statistical analysis. A face chart was given to each subject after resolution of the glutamate-evoked pain to draw the area of perceived pain on the chart. VistaMetrix for quantitative data extraction from graphics (SkillCrest, LLC, version 1.36, LEAD Technologies, Inc., Tucson, AZ, USA) was used to calculate the area in arbitrary units.

##### Skin Temperature

Prior to and after each intramuscular injection, vasomotor responses reflected as surface temperature on the skin overlying the temporalis muscles were assessed by a non-contact infrared thermographic camera (FLIR Systems AB, Täby, Sweden). The temperature resolution of the device is 0.09 °C. The distance between the camera lens and the tissue was set to 50 cm and measurements were performed at baseline and 5, 15 and 30 min after each injection. To gain the profile and magnitude of local changes of the temperature, a region of interest (ROI) was defined with a circular shape (area ≈ 2.5 cm^2^) where the injection site was located at the center of the region. Thermographic images were stored on the computer’s hard disk for off-line analysis. The average temperature within the ROI was then calculated by ThermaCAM researcher Pro 2.8 (FLIR Systems AB, Täby, Sweden) and used for statistical analysis. Measurements were performed in a temperature controlled and semi dark room to eliminate artifact from ambient room lighting.

##### Skin Blood Perfusion

In parallel with the thermography, blood perfusion on the skin overlying the temporalis muscles was measured by a non-contact laser Doppler imager (LDI2, Moor Instruments, Devon, UK). Laser Doppler imaging is a sophisticated scanning technique for non-invasive monitoring of microvascular perfusion often referred to as microvascular blood flow or red blood cell flux. An area of 3.0 × 4.0 cm^2^ was scanned at a distance of 30 cm from the skin. The image resolution was obtained at 118 × 70 pixels with a speed of 4 ms/pixel. Each scan lasted 1 min. Bandwidth was set at 250 Hz to 15 Hz. The blood flux recordings were performed at baseline and 5, 15 and 30 min after each injection. To gain the profile and magnitude of local changes of the blood perfusion, a ROI was defined with a circular shape (area ≈ 2.5 cm^2^) where the injection site was located at the center of the region. The scans were stored on computer’s hard disk for off-line analysis. Average blood perfusion within the ROI was calculated by designated Moor software (v5.3) (expressed in arbitrary units) and used for statistical analysis. Measurements were performed in a temperature controlled and semi dark room to eliminate artifact from ambient room lighting. To protect the subject’s eyes, special goggles were provided.

#### 3.1.6. Statistical Analysis

Based on our results from a previous study on a capsaicin-evoked pain model and the analgesic effect of BoNTA [35], a sample size of 30 subjects (15 from either sex) was considered sufficient to detect the BOTOX^®^ suppressive effect in the present study. The desired significance level (*α*) was set to 0.05 by convention and the desired power (1-*β*) was set to 0.8, acknowledging the importance sufficient power in comparison studies. The clinically relevant effect (*E*) was estimated as 30% of the change from baseline to post BoNTA treatment. Data are presented as mean and standard error of the mean (mean ± SEM) in text and figures, unless otherwise specified. Data were analyzed with multi factor repeated measures ANOVA (RM ANOVA). For the glutamate-evoked pain response factors were defined as treatment (glutamate and saline), time (baseline, 5, 15 and 30 min) and sex (male and female). Likewise for the BoNTA analgesic effect a total of three factors were used and defined as follows: treatment (BoNTA and saline), time (3 h, 7, 30 and 60 days) and sex (male and female). Except for all the pain characteristics, where the 2-way RM ANOVA was used, the 3-way RM ANOVA was applied for all the remaining analysis. Bonferroni test was used for *post hoc* analysis. All statistical tests were carried out by IBM SPSS, version 20 and the level of significance was set at P < 0.05.

## 4. Conclusions

The present study demonstrated that the onset of analgesia after intramuscular injection of BoNTA may occur within a few hours, rather than after days as previously described [38]. This effect, at least in part, might be due to direct action of BoNTA on peripheral nociceptors that results from its ability to block vesicular release of neuroactive substances. The present data indicated that BoNTA decreased glutamate-induced pain and vasomotor reactions, but sex-related differences were limited to vasomotor responses. These data suggest that it may be possible to monitor the onset and efficacy of BoNTA when used as an analgesic agent through the use of non-invasive techniques such as thermal imaging; however, adjustments may be required to account for sex-related differences in these measures.
